# Diverse functions and pathogenetic role of Crumbs in retinopathy

**DOI:** 10.1186/s12964-024-01673-z

**Published:** 2024-05-27

**Authors:** Xuebin Zhou, Liangliang Zhao, Chenguang Wang, Wei Sun, Bo Jia, Dan Li, Jinling Fu

**Affiliations:** 1 Department of Ophthalmology, The Second Hospital of Jilin University, Changchun, 130000 China; 2 College of Basic Medical Sciences, Jilin University, Changchun, 130000 China

**Keywords:** Crumbs, Retinopathy, Animal model, Cell polarity, Adherens junction

## Abstract

The Crumbs protein (CRB) family plays a crucial role in maintaining the apical–basal polarity and integrity of embryonic epithelia. The family comprises different isoforms in different animals and possesses diverse structural, localization, and functional characteristics. Mutations in the human *CRB1* or *CRB2* gene may lead to a broad spectrum of retinal dystrophies. Various CRB-associated experimental models have recently provided mechanistic insights into human CRB-associated retinopathies. The knowledge obtained from these models corroborates the importance of CRB in retinal development and maintenance. Therefore, complete elucidation of these models can provide excellent therapeutic prospects for human CRB-associated retinopathies. In this review, we summarize the current animal models and human-derived models of different CRB family members and describe the main characteristics of their retinal phenotypes.

## Background

Cell polarity governs several critical cellular processes, such as cell proliferation, migration, differentiation, vesicle trafficking, and immune responses [1, 2]. It is an essential and pivotal property from bacteria to eukaryotic cells [3, 4]. Polarity regulators are traditionally subdivided into three protein complexes: Crb, Par, and Scribble, initially identified in *Drosophila melanogaster* and *Caenorhabditis elegans* [5]. There is evidence of numerous interactions among these proteins to delicately balance apical, lateral, and basal polarity rather than functioning discretely [5, 6].

The Crb complex is highly conserved and thought to play a vital role in maintaining apical–basal polarity and integrity of embryonic epithelia [7, 8], the central constituent of which, the CRB protein, contains isoforms different across animals. For example, in *Drosophila*, there is only one Crb type [7, 8], but mammals have three isoforms: CRB1, CRB2, and CRB3 [9], and zebrafish has five: Crb1, Crb2a (oko meduzy, ome), Crb2b, Crb3a, and Crb3b [10].

In humans, CRB1 has more retinal functions than CRB2 and CRB3. Mutations in *CRB1* often lead to a wide spectrum of retinal dystrophies, such as retinitis pigmentosa type 12 (RP12), Leber congenital amaurosis type 8 (LCA8), pigmented paravenous chorioretinal atrophy, and retinitis pigmentosa with coat’s-like exudative vasculopathy [11–14]. Variants of human *CRB2* have recently been linked to reduced visual acuity, irregular retinal pigmentation, and mild optic atrophy [15, 16]. However, no known human diseases are associated with *CRB3* mutations [17].

In this review, we summarize the mutations and alterations in the *CRB* gene in current animal models and human-derived models, as well as describe the main characteristics of their retinal phenotypes. These models contribute to gaining mechanistic insight into CRB protein functions and developing treatments for CRB-associated retinopathies.

## CRB structure

**Fig. 1:**
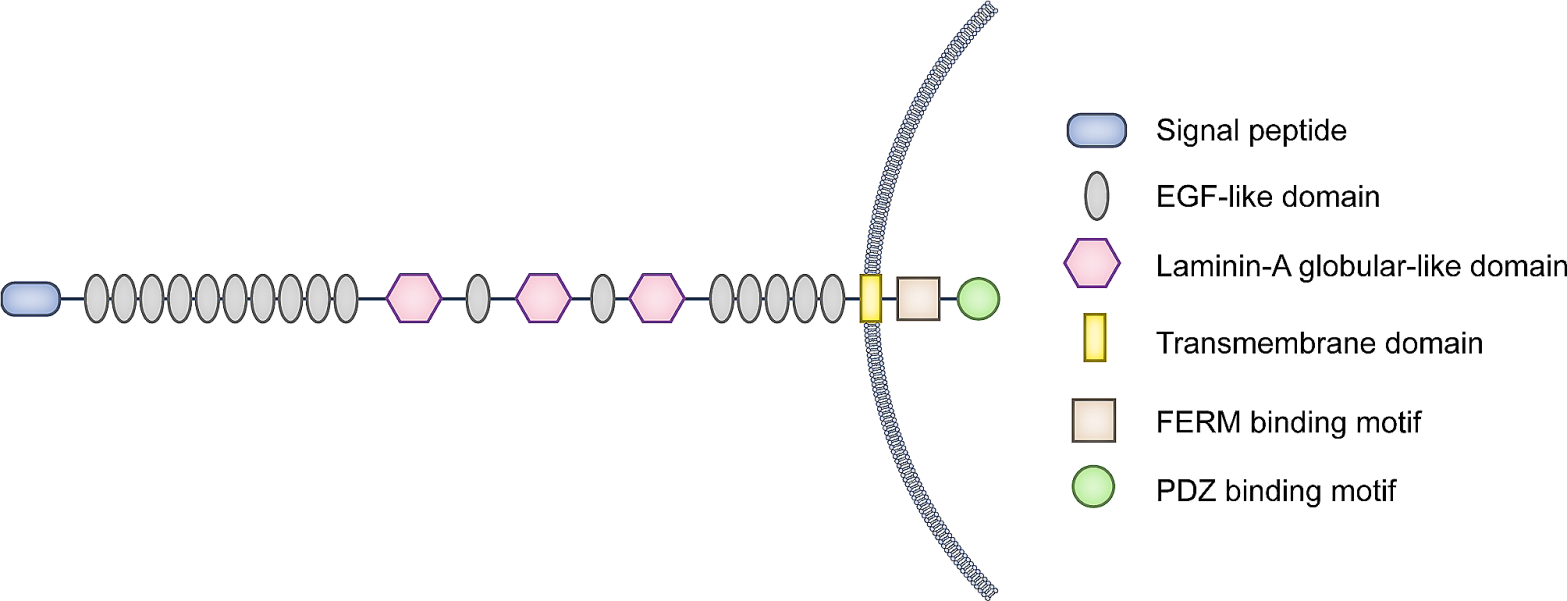
Schematic diagram of the structure of the prototypic Crumbs protein. CRB protein is a type I transmembrane protein with large extracellular, single transmembrane, and small intracellular domains. The large extracellular N-terminal domain includes a signal peptide sequence, multiple EGF-like repeats, and several laminin-A globular-like repeats. The small but essential intracellular domain contains two highly conserved elements: a juxtamembrane FERM-binding motif and a C-terminal PDZ-binding motif

The core Crb complex is composed of CRB protein, Protein Associated with Lin Seven 1 (PALS1), PALS1-associated tight junction protein (PATJ), and Multi-PDZ domain Protein 1 (MUPP1) [18–20]. The prototypic CRB protein is a type I transmembrane protein with large extracellular, single transmembrane, and small intracellular domains. The large extracellular N-terminal domain includes a signal peptide sequence, multiple epidermal growth factor (EGF)-like repeats, and several laminin-A globular-like repeats. The small but essential intracellular domain contains two highly conserved elements: a juxtamembrane FERM-binding motif and a C-terminal PDZ-binding motif [7] (Fig. 1). The FERM motif binds to Erythrocyte Protein Band 4.1-like 5 (EPB4.1L5) in mammals (Yurt in *Drosophila*, Mosaic Eyes in zebrafish) [21–23]. The PDZ motif binds to the PDZ domain of PALS1, which further binds to PATJ and MUPP1 *via* its N-terminal L27 domain [24].

More recently, Ray et al. [25]. used the long-read capture sequencing (lrCaptureSeq) strategy to show that *CRB1* in vertebrate species is a group of variants bearing unconventional 5′ and 3′ ends. The canonical transcript, *CRB1*-A, encodes a 1406-amino acid protein with highly conserved EGF-like and laminin-A globular-like domains. The *CRB1*-B transcript encodes a 1003-amino acid protein that lacks the conserved motif. The third transcript, *CRB1*-C, encodes a 754-amino acid protein without transmembrane and intracellular domains. These findings indicate the genetic and pathobiological roles of CRB1 isoforms in CRB-associated retinopathies.

### Localization of CRB in the retina

Generally, CRB proteins reside in the subapical regions (SARs) adjacent to the adherens junctions (AJs) between Müller glial cells (MCs), photoreceptor cells (PRCs), MCs–MCs, and PRCs–PRCs [16, 24, 26–29]. However, its localization also shows unique temporal and spatial characteristics across species and developmental stages.

**Fig. 2:**
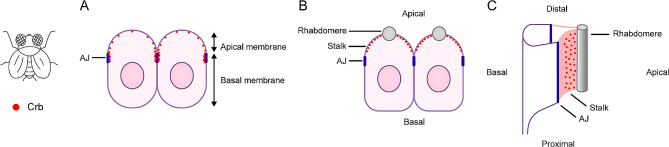
Schematic diagram of the localization of Crumbs proteins in the *Drosophila* retina. **(A)** In early *Drosophila* embryos, Crb is distributed throughout the apical membrane but is concentrated at the cell boundaries adjacent to AJs. **(B, C)** When the rhabdomere and stalk domains are formed morphologically, Crb is exclusively distributed in the stalk membrane but not in the vicinity of the AJs.

In early *Drosophila* embryos, Crb is distributed throughout the apical membrane but is concentrated at the cell boundaries adjacent to AJs. When rhabdomere and stalk domains are morphologically formed, Crb is the exclusive component of the stalk membrane. However, Crb is not present in the vicinity of the AJs. This specific stalk localization of Crb is maintained in adult *Drosophila* [30] (Fig. 2).

**Fig. 3:**
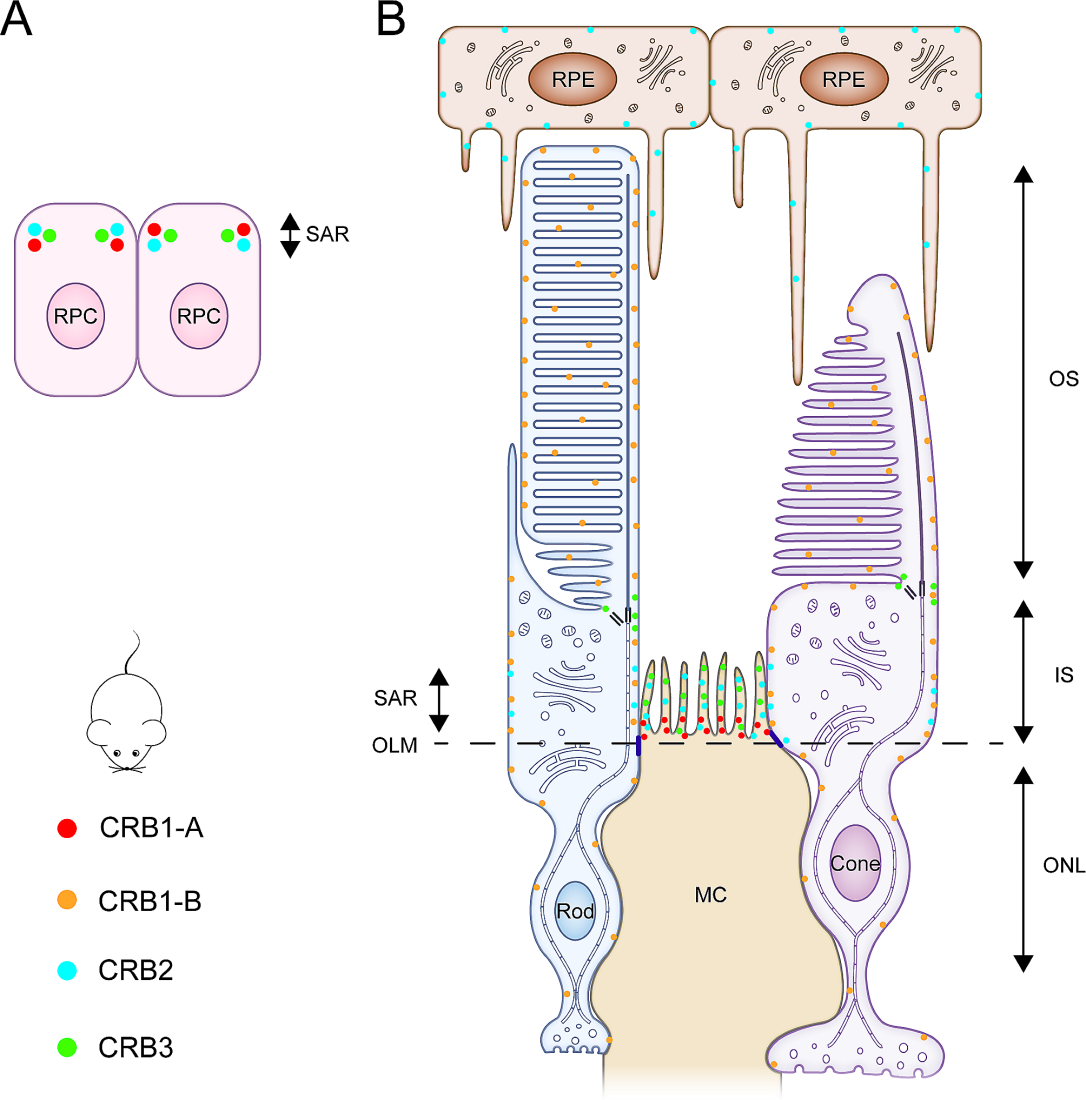
Schematic diagram of the localization of Crumbs proteins in the mouse retina. Mouse has a specific expression pattern of CRB1: CRB1-A is predominant during development, and CRB1-B is the most abundant isoform in the adult retina. **(A)** CRB1-A, CRB2, and CRB3 localize to the SAR of RPC. **(B)** In the adult mouse retina, CRB1-A is expressed primarily by MCs and selectively localizes to the OLM junctions. CRB1-B is distributed throughout the PRCs, mainly concentrated in the IS and OS. CRB2 is present in the RPE and SAR of MCs and PRCs. CRB3 is distributed in the microvilli of MCs and IS of PRCs and is particularly concentrated in cilia-connecting regions. In addition, CRB3 is present at the synaptic terminals of the outer plexiform layer and bipolar and amacrine cells of the inner plexiform layer (not shown)

Mouse studies have detected a cell-type-specific expression pattern of *CRB1*-A and *CRB1*-B: CRB1-A is present primarily in MCs and selectively localizes to outer limiting membrane (OLM) junctions, whereas CRB1-B is distributed throughout PRCs, mainly concentrated in the inner segments (IS) and outer segments (OS). CRB1-A is predominant during development, and CRB1-B is the most abundant isoform in the adult retina [25]. CRB2 is present in the adult mouse retinal pigment epithelium (RPE) and the SARs of MCs, PRCs, and retinal progenitor cells (RPCs) [31–33]. CRB3 is distributed in the microvilli of MCs and IS of PRCs, particularly in cilia-connecting regions (Fig. 3). In addition, CRB3 is present at the synaptic terminals of the outer plexiform layer and bipolar and amacrine cells of the inner plexiform layer [26, 34].

**Fig. 4:**
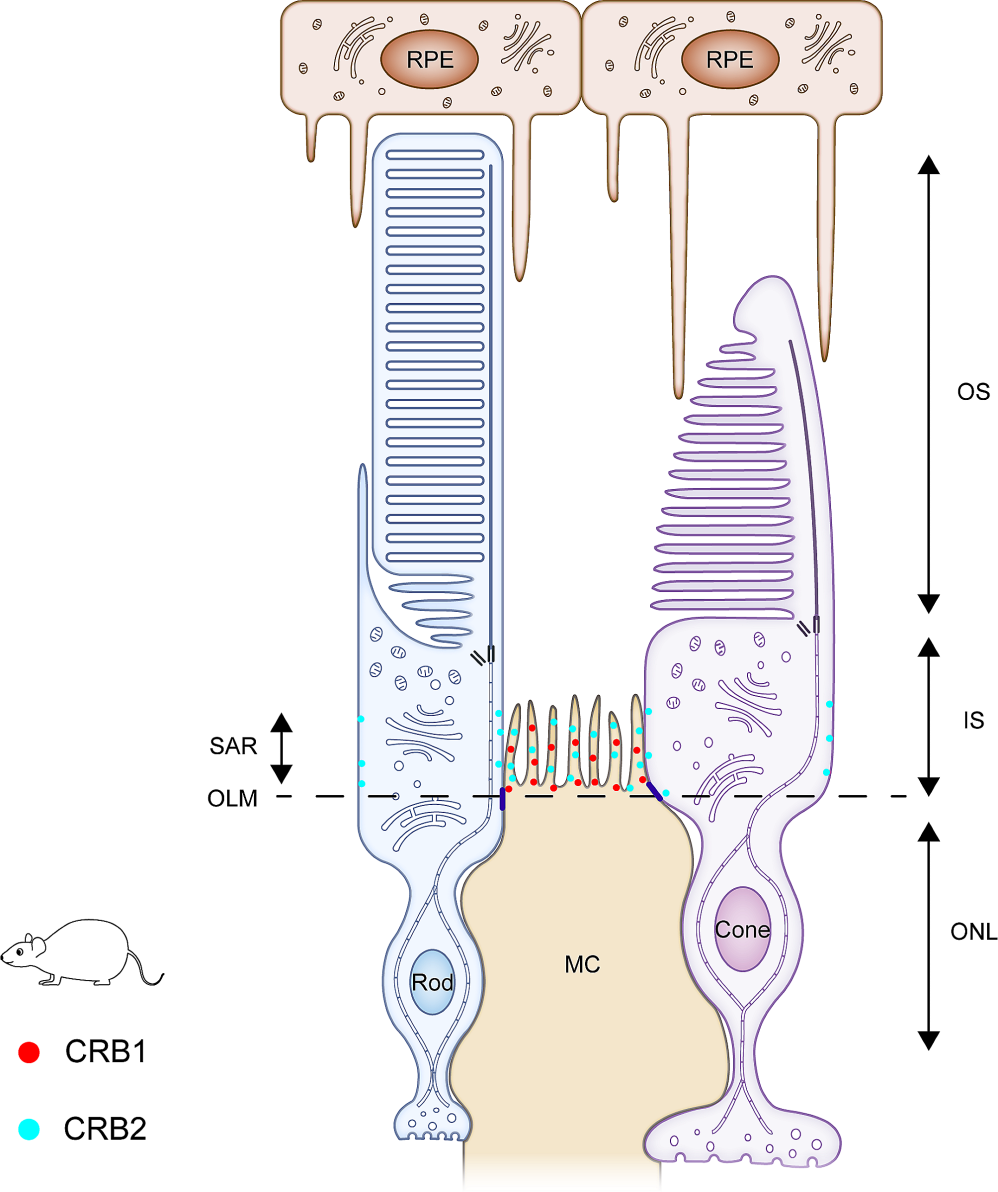
Schematic diagram of the localization of Crumbs proteins in the rat retina. According to the results of immuno-electron microscopy, CRB1 is abundantly present at the SARs in MCs but not PRCs, while CRB2 is present at the SARs of both MCs and PRCs. There are few reports about the distribution of CRB3 in the rat retina

The cellular localizations of CRB1 and CRB2 in rat are approximately similar to those in mouse: according to the results of immuno-electron microscopy, CRB1 is abundantly present at the SARs in MCs but not PRCs, while CRB2 is present at the SARs of both MCs and PRCs [35] (Fig. 4). There is no report about the distribution of CRB3 in the rat retina.

**Fig. 5:**
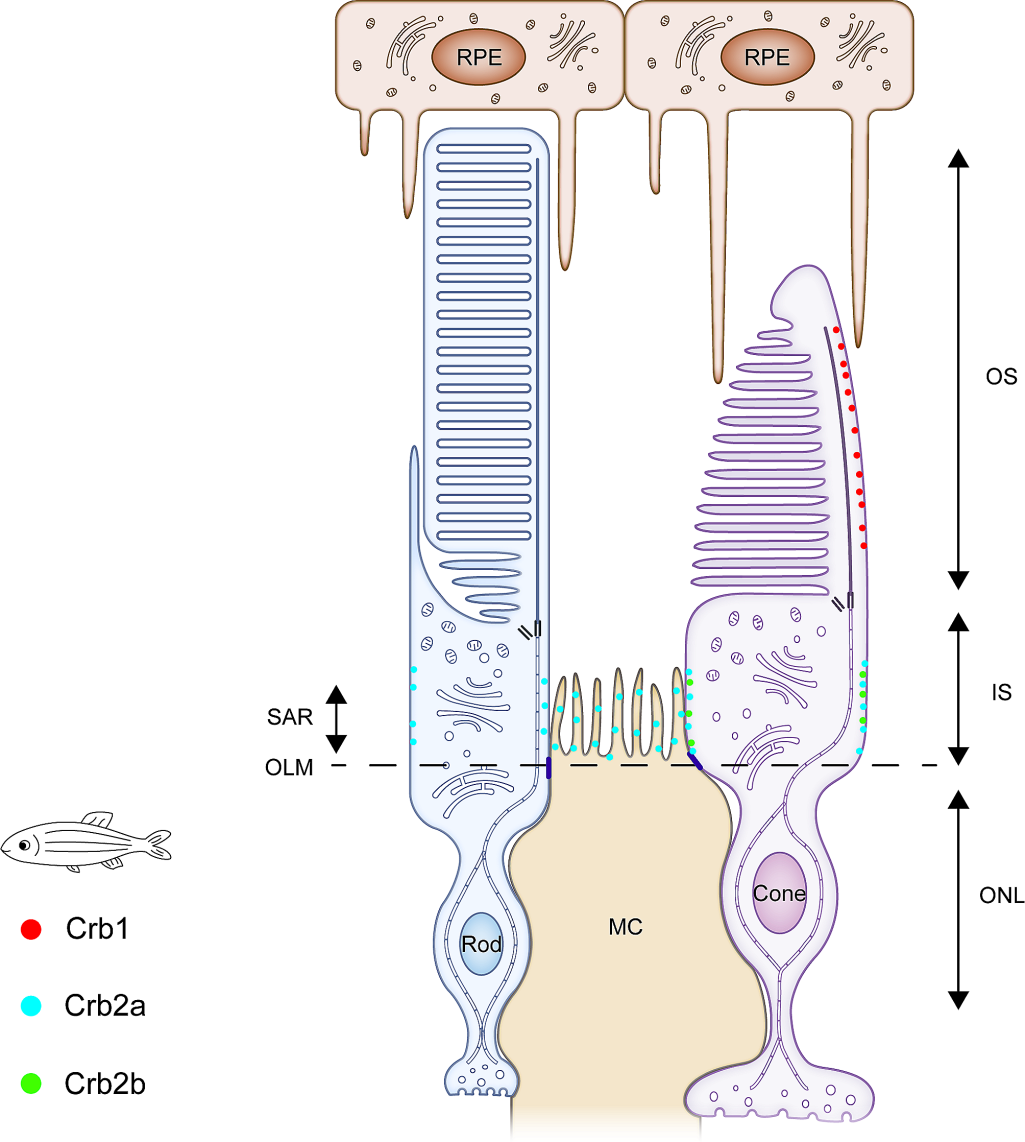
Schematic diagram of the localization of Crumbs proteins in the zebrafish retina. Zebrafish has five isoforms of Crb: Crb1, Crb2a, Crb2b, Crb3a, and Crb3b. Crb1 is present in the cone OS surrounding the axonemes. Crb2a is present at the SAR of the MCs and PRCs, adjacent to the OLM and away from axonemes. Crb2b is limitedly present at the SAR of RGB cones, adjacent to the OLM and away from the axonemes. Crb3a and Crb3b are not expressed in the zebrafish retina

The expression pattern of *Crb* in zebrafish is different from that in other vertebrates: Crb1 is present in all types of cones but not rods; Crb2a is present in all MCs and PRCs of the developed retina and undifferentiated retinal neuroepithelial cells; Crb2b is limitedly present in RGB cones; and Crb3a and Crb3b are not present in the zebrafish retina. Specifically, MCs and rods only express Crb2a, all cones express Crb1 and Crb2a, RGB cones can also express Crb2b, and other retinal cells do not express Crb [36–40]. As for subcellular localization, Crb2a and Crb2b are conventionally located in the SARs of MCs and/or PRCs, adjacent to the OLM and away from axonemes [36, 40]. However, zebrafish Crb1 is specifically localized in the cell membranes surrounding axonemes in the OS of the cones [40] (Fig. 5).

**Fig. 6:**
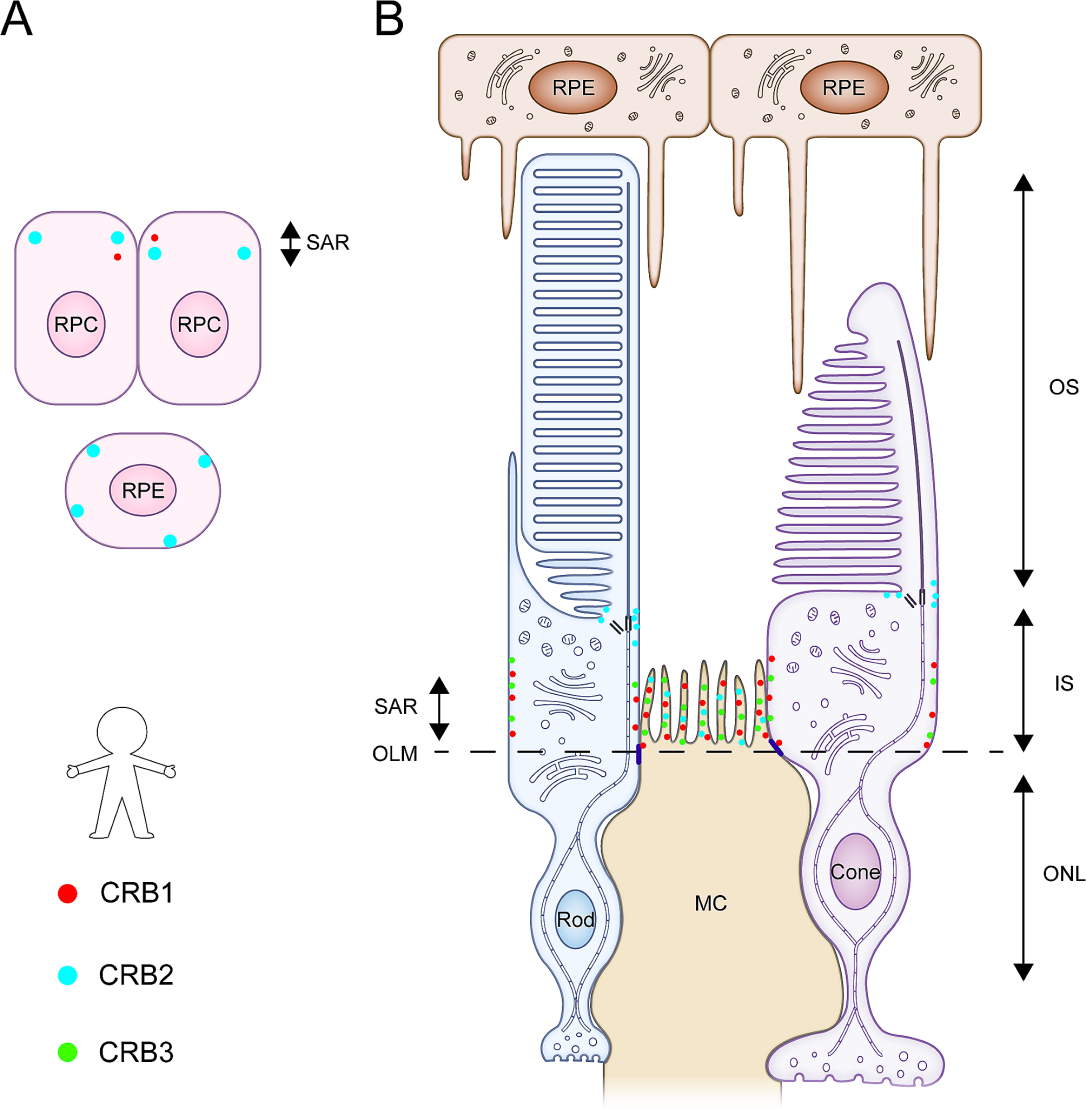
Schematic diagram of the localization of Crumbs proteins in the human retina. Human retinas exhibit a unique expression and localization pattern of CRB proteins. **(A)** In the first trimester of the human fetal retina, CRB1 and CRB2 are present in SARs adjacent to AJs in RPCs, but the distribution of CRB1 is low and sporadic. In the first and second trimesters, CRB2 is also expressed in the human fetal RPE. **(B)** In the mid-second trimester of the human fetal retina, CRB1 and CRB2 labeling are detectable in SARs adjacent to AJs between putative PRC IS and in the apical villi of radial glial progenitor cells/MCs, similar to their distribution pattern in the adult retina. In the adult human retina, CRB1 and CRB3 are present at the SAR of MCs and PRC IS, whereas CRB2 is present at the SAR of MCs and vesicles in the PRC IS but at some distance from the SAR.

Human retinas exhibit a unique expression and localization pattern of CRB proteins. In the first trimester of the human fetal retina, CRB2 is present in SARs adjacent to AJs in RPCs, whereas the distribution of CRB1 is low and sporadic. Subsequently, in the mid-second trimester of the human fetal retina, CRB1 and CRB2 labeling are detectable in SARs adjacent to AJs between putative PRC IS and in the apical villi of radial glial progenitor cells/MCs [41]. CRB2 is also present in the first and second trimesters of human fetal RPE [42]. Finally, in the adult human retina, CRB1 and CRB3 are present in SARs of MCs and PRC IS, whereas CRB2 is present in SARs of MCs and the vesicles in the PRC IS but at some distance from the SARs [41] (Fig. 6). Recently, Stehle et al. [43]. confirmed by immunohistochemistry that canonical CRB1 and CRB2 co-localize at the OLM in human induced pluripotent stem cell (iPSC)-derived retinal organoids and adult human donor retina. Co-immunoprecipitation assays further demonstrated the homotypic and heterotypic interactions between canonical CRB1 and CRB2 through their large extracellular domains. These data provide a first hint on a possible interdependence of canonical CRB1 and CRB2. CRB1 and CRB2 homomerization and heteromerization may directly contribute to the establishment and maintenance of OLM.

Similar localizations have been found in the retinas of rhesus and cynomolgus macaques. CRB1, CRB2, and CRB3a are present in SARs in MC apical villi and PRC IS. CRB3a can also be detected in the RPE in macaques [44].

### Current models of CRB-associated retinopathies

Early studies in *Drosophila* have described the properties of Crb in controlling the integrity of AJs and the formation of rhabdomeres, which may provide some clues to the pathogenesis of human CRB-associated retinopathies [30, 45]. However, *Drosophila* models are restricted by their different retinal morphologies and developmental patterns, compared to vertebrate models. As a result, an increasing number of rodent and zebrafish models have been used to investigate the functions of CRB proteins in the retina. More recently, human-derived models provide direct guidance for evaluating biological activity. In this section, we summarize the current models of different CRB subtypes and describe the main characteristics of their retinal phenotypes.

### Mouse models mimicking CRB-associated retinopathies

#### Mouse retina lacking CRB1

Three mouse models lack CRB1 in the retina. First, mice with natural mutations in *CRB1* are referred to as *rd8* mice (*Crb1*^*rd8*^ mice) [29]. *Crb1*^*rd8*^ mice have a single-base deletion, which is predicted to result in a frameshift mutation and premature stop codon, truncating the transmembrane and cytoplasmic domains of the CRB1 protein [29]. The IS and OS of PRCs were shortened in 2-week-old *Crb1*^*rd8*^ mice, suggesting a developmental defect. PRC degeneration was observed only in dysplastic spotted regions with retinal folds and pseudorosettes, mainly in the inferior nasal quadrant of the retina [29]. Although *Crb1*^*rd8*^ mice showed patchy retinal disorganization, there was sufficient photoreceptor integrity to produce essentially normal retinal functions [46]. It is known that C57BL/6 N mice harbor *Crb1*^*rd8*^ mutation. In contrast, C57BL/6J mice might have unknown genetic modulators of *Crb1*^*rd8*^, therefore the phenotype of photoreceptor dysplasia and degeneration is strongly suppressed in the C57BL/6J genetic background [29]. Previous studies suggest that *Crb1*^*rd8*^ mutation, as a gene modifier, has significant influences on many experimental models of retinal pathology studies. When *Crb1*^*rd8*^ coexists with certain gene mutations, it tends to support earlier onsets and worsened retinal alterations [47–49]. Nuclear factor E2-related factor 2 knock out (*NRF2*^*−/−*^) mice lack antioxidant mechanisms, which develop an age-related macular degeneration (AMD) phenotype with drusen-like retinal spots, increased Bruch’s membrane thickness, and may even develop choroidal neovascularization (CNV) [50, 51]. Homozygous *Crb1*^*rd8/rd8*^ mutation can increase the severity of AMD-like retinal alterations and favor CNV formation in *NRF2*^*−/−*^ mice [47]. Methylenetetrahydrofolate reductase (MTHFR) is a key enzyme in homocysteine-methionine metabolism [52]. *Mthfr*^*+/−*^ mouse is a murine model of mild hyperhomocysteinemia, which shows decreased ganglion cell function, thinning of nerve fiber layer, and mild retinal vasculopathy [53]. The *Mthfr*^*+/−*^ mice bred on *Crb1*^*rd8*^ background support the notion of accelerated and aggravated retinal dysfunction. The combined mutants even present an intriguing “geographic atrophy” which is not a prominent finding of either *Mthfr*^*+/−*^ mice or *Crb1*^*rd8*^ mice [49]. Cytoglobin (Cygb) is an evolutionary ancient heme-containing protein with poor functional annotation [54]. There are no obvious retinal abnormalities in the *Cygb* knock out (*Cygb*^*−/−*^) mice. However, the absence of Cygb on the *Crb1*^*rd8*^ background worsens the photoreceptor lamination defects and accelerates the retinal degeneration seen in *Crb1*^*rd8*^ mice [48]. Prior investigation of the *Crb1*^*rd8*^ background in ocular experimental models is essential to preempt potential confusion in retinal science research.

The second phenotype of *CRB1* mutant mice harbors an amino acid missense mutation (Cys249Trp) in the sixth extracellular calcium-binding EGF region, named *Crb1*^*C249W*^ mice, which served as a putative mouse model for RP [27]. The truncated *Crb1*^*C249W*^ protein was properly produced and localized to SARs adjacent to AJs [27]. *Crb1*^*C249W*^ mice showed significant retinal degeneration at 18 months (M) of age and onwards [27]. Loss of PRCs was observed at 18 M and 24 M, whereas loss of retinal electrical activity was still undetectable at 18 M or 30M [27]. This late onset of the retinal phenotype may be due to different genetic backgrounds or the redundancy of CRB proteins. Light irradiation increased the incidence of retinal folds in *Crb1*^*C249W*^ mice [27]. In addition, it significantly downregulated the transcriptional levels of retinal pituitary tumor transforming gene 1 (*Pttg1*) [27]. *Pttg1* is a securin originally found in pituitary tumors [55]. It can inhibit the premature segregation of sister chromatids during mitosis and has transcriptional activity in cell proliferation, apoptosis, and transcription [56–58]. These studies suggest that the extracellular domain of CRB1 regulates cell death or apoptosis by influencing the transcriptional level of *Pttg1*. In addition, other proteins that interact with CRB1 may participate in the CRB1/Pttg1 signaling pathway.

In *CRB1* knockout (*Crb1*^*−/−*^) mice, the promoter and the first exon encoding the N-terminus of CRB1 were deleted. Complete ablation of CRB1 in mice might result in an PR-like phenotype, initially showing a normal retina but developing into localized lesions by 3–9 M, in which the integrity of the OLM was impaired, and giant half rosettes or double PRC layers were observed [28]. After photoreceptor layer delamination, neuronal cell death occurs in the inner nuclear layer (INL) and outer nuclear layer (ONL) of the retina [28]. This disorganization mainly occurred in the inferior temporal quadrant and showed a light-accelerated characteristic [28, 59]. Light exposure also alters the expression of other genes involved in chromosome stabilization [59]. Therefore, it can be considered that CRB1 is essential for the maintenance, rather than assembly, of AJs between MCs and PRCs and that light may be an influential factor in the development of retinopathies.

#### Mouse retina lacking CRB2

The deficiency of mouse CRB2 can affect gastrulation, resulting in embryonic lethality [60]. To evaluate the function of CRB2 in the mouse retina, *loxP* sites were inserted in the *CRB2* between exons 9a and 10 and in exon 13 downstream of the stop codon to generate *CRB2* floxed homozygous (*Crb2*^*flox/flox*^) mouse [31]. Chx10-*Cre* drives CRE-mediated recombination in the neuroepithelial RPCs [61]. Crossing the *Crb2*^*flox/flox*^ mouse and Chx10-*Cre* mouse to obtain *Crb2*Chx10 conditional knockout (cKO) line resulted in the loss of *CRB2* expression at the apical surface from embryonic day (E) 12.5 [31]. Removal of mouse CRB2 from RPCs and consequent removal from PRCs and MCs resulted in abnormal lamination throughout the retina and progressive attenuation of retinal function, similar to RP. Histologically, AJs between MCs, PRCs, and RPCs were disrupted, ectopic PRC nuclei were observed in the space between the RPE and OLM, and the OS of the cones became shorter. In addition, the number of late-born RPCs, rods, and MCs was increased, concomitant with programmed cell death of rods, suggesting that CRB2 plays an essential role in proper retinal lamination and suppression of late-born RPC proliferation. Loss of CRB2 also led to the disorganization of other apical proteins, such as MUPP1, PALS1, and Par3, indicating that retinal CRB2 is required to stabilize the CRB complex and its interacting complexes. Contrary to observations in *Crb1*^*rd8*^ or *Crb1*^*−/−*^ mice, the retinal phenotype of *Crb2*Chx10 cKO mice was not suppressed in the C57BL/6J genetic background [62].

The Crx-*Cre* mice express CRE recombinase in immature PRCs from E12.5 [63, 64]. Crossing *Crb2*^*flox/flox*^ mouse with Crx-*Cre* mouse to obtain mice with targeted deletion of CRB2 in both immature cones and rods (*Crb2*^*ΔimPRC*^ mice). The *Crb2*^*ΔimPRC*^ retinas also mimicked RP, which resulted in an early, severe, and progressive retinal phenotype throughout the retina, with concomitant functional impairment [65].

The Rhodopsin-*iCre* mouse express CRE recombinase in rods from postnatal day (P) 7, resulting in efficient recombination at P18 [66]. Crossing *Crb2*^*flox/flox*^ mouse with the Rhodopsin-*iCre* mouse to obtain rod-specific CRB2 ablation (*Crb2*^*ΔRods*^) mice. The *Crb2*^*ΔRods*^ retinas preserved gross retinal lamination, mainly resulting in the loss of rods and gliosis in the central superior and peripheral quadrants, similar to RP. Rod function was impaired in 9M *Crb2*^*ΔRods*^ mice [67].

In Pdgfrα-Cre mouse lines, CRE expression is directed to nearly all MCs by mouse Pdgfrα (platelet-derived growth factor receptor, alpha polypeptide) promoter [68]. Therefore, specific removal of CRB2 from MCs could be achieved by crossing *Crb2*^*flox/flox*^ mouse with Pdgfrα-Cre mouse to obtained *Crb2*Pdgfrα cKO mice (abbreviated as *Crb2*^*ΔMG*^ mice). This group showed a mild retinal phenotype, primarily affecting the periphery of the retina, with slow progression and undetectable functional consequences measured by electroretinography (ERG) [65].

Moreover, Alves et al. [65]. transferred AAV vectors encoding CRE recombinase and *short hairpin RNA* against *CRB2* into C57BL/6J retinas to reduce *CRB2* expression, providing valuable insights into the phenotype and the role of CRB2 in the adult mouse retina. In the adult retina, short-term depletion of CRB2 in PRCs led to sporadic loss of AJs between PRCs or MCs, suggesting that CRB2 is required for the maintenance of retinal structure. However, short-term depletion of CRB2 in MCs did not affect the retina integrity, which could be explained in part by the CRB1 compensation in MCs.

#### Mouse retina lacking CRB1 and CRB2

*Crb1*^*−/−*^ mice were crossed with *Crb2*Chx10 cKO mice to obtain *Crb1*^*+/−*^*Crb2* cKO and double homozygote *Crb1Crb2* cKO [69]. The lack of CRB1 and CRB2 severely impaired retinal function, disrupted retinal lamination, and transiently increased retinal thickness at P10 and P14, followed by progressive thinning and degeneration [69]. Thickening of the retina was attributed to RPCs undergoing several more cell cycles than control retinas, indicating over-proliferation during late retinal development, leading to an increased number of late-born retinal cells, such as rods, MCs, and bipolar cells [69]. This uncontrolled proliferation of RPCs was putatively caused by the downregulation of Notch1 and Hippo/YAP signaling and increased levels of P120-catenin [69]. Ablation of CRB1 and CRB2 mimicked the LCA, whereas the retinas in the single heterozygote *Crb1*^*+/−*^*Crb2* cKO displayed intermediate phenotypes between the *Crb2*Chx10 cKO and the double homozygote *Crb1Crb2* cKO [31, 69].

Deletion of one allele of *CRB2* in heterozygote *Crb1*^*+/−*^ and homozygote *Crb1*^*−/−*^ mouse retinas generated double heterozygote *Crb1*^*+/−*^*Crb2*^*F/+*^ cKO and single heterozygote *Crb1Crb2*^*F/+*^ cKO, respectively [26]. The *Crb1*^*+/−*^*Crb2*^*F/+*^ cKO retinas showed mild disorganization starting at 3 M and no change in retinal activity up to 12 M. The phenotype increased with exposure to constant white light for 72 h [26]. In contrast, the first disorganization in the *Crb1Crb2*^*F/+*^ cKO retina was observed early at 10 days, followed by rapid and severe degeneration of the whole inferior retina at 3 M, accompanied by impairment of retinal activity [26]. In addition, data showed that inferior retinas were more likely to be affected in *Crb1*^*rd8*^ [29], *Crb*^*−/−*^ [28], and *Crb1Crb2*^*F/+*^ cKO retinas [26]. This feature may be related to the uneven distribution of CRB1 and CRB2 proteins between the inferior and superior retinas (in wild-type mice, *CRB1* was highly expressed in the superior retina but substantially lower in the inferior retina, whereas *CRB2* was highly expressed in the inferior retina but substantially lower in the superior retina) [26].

Conditional disruption of one allele of *CRB2* in PRCs on a *Crb1*^*−/−*^ genetic background (*Crb1*^*KO*^*Crb2*^*Low − immPRC*^ mice) produced an RP-like phenotype, whereas conditional full ablation of CRB2 in PRCs on a *Crb1*^*−/−*^ genetic background (*Crb1*^*KO*^*Crb2*^*ΔimmPRC*^ mice) resulted in a phenotype similar to that of LCA [70]. *Crb1*^*KO*^*Crb2*^*Low − immPRC*^ mice showed extensive lamination defects and PRC death in the inferior retina with concomitant retinal function impairment, which was significantly more severe than that of retinas that lacked CRB1. The phenotype of *Crb1*^*KO*^*Crb2*^*ΔimmPRC*^ retina was much more aggravated but surprisingly more severe on the superior than on the inferior side [70]. These data suggest that the remaining CRB2 proteins in MCs and RPCs are insufficient to maintain AJs at the OLM between various retinal cells, and the opposite gradients of *CRB1* and *CRB2* expression may be responsible for the superior–inferior symmetry of mouse retinas [70].

The *Crb1*^*KO*^*Crb2*^*ΔRods*^ mouse is a model of targeted ablation of CRB2 in rods on the *Crb1*^*−/−*^ genetic background, resulting in an early and aggravated RP phenotype, predominantly in the peripheral and central superior retina, similar to that in the *Crb2*^*ΔRods*^ retinas [67]. In addition, a moderate reduction in the amplitudes of a-wave scotopic ERG responses and a substantial reduction in contrast sensitivity could be observed from 3 M onwards [67].

Crossing *Crb1*^*−/−*^ mice with *Crb2*^*ΔMG*^ mice generated *Crb1*^*KO*^*Crb2*^*ΔMG*^ mice with a specific deletion of CRB2 in MCs on the genetic background of CRB1 deletion [44]. The remaining CRB2 levels in PRCs and radial glial progenitors were insufficient to maintain the lamination and function of the *Crb1*^*KO*^*Crb2*^*ΔMG*^ retina, resulting in an LCA-like phenotype [44]. Morphological alterations could be detected at E17.5 in the peripheral retina and showed severe and progressive retinal dystrophy from the peripheral to the central retina with associated dysfunction [44]. Disruption of the OLM contributes to the ectopic localization of RPCs, PRCs, and bipolar cells [44].

Reducing endogenous mouse *CRB2* expression in MCs from one instead of two *CRB2* alleles in *Crb1*^*−/−*^ mice generated a less severe mouse model: *Crb1*^*KO*^*Crb2*^*LowMGC*^ [71]. Reduced endogenous CRB2 levels in MCs on the genetic background of CRB1 deletion results in a RP retinal phenotype with foci of retinal disorganization mostly in the two inferior quadrants [71]. Morphological and functional alterations could be detected from 3 M onwards and worsened over time. Furthermore, *Crb1*^*KO*^*Crb2*^*LowMGC*^ showed increased susceptibility to OLM disruptions upon exposure to DL-α-aminoadipate acid (DL-AAA, an MC-specific toxin that decreases the reserve pool of cysteine and glutathione in MCs) [71–73].

Taken together, several mouse lines exhibit the RP-like phenotype: *Crb1*^*rd8*^, *Crb1*^*C249W*^, *Crb1*^*−/−*^, *Crb2*Chx10 cKO, *Crb2*^*ΔimPRC*^, *Crb2*^*ΔRods*^, *Crb2*^*ΔMG*^, *Crb1*^*+/−*^*Crb2*^*F/+*^ cKO, *Crb1Crb2*^*F/+*^ cKO, *Crb1*^*KO*^*Crb2*^*Low − immPRC*^, *Crb1*^*KO*^*Crb2*^*ΔRods*^, and *Crb1*^*KO*^*Crb2*^*LowMGC*^ mice. Four mouse models show a more severe phenotype similar to LCA: (i) *Crb1*^*+/−*^*Crb2* cKO with reduced level of CRB1 and absence of CRB2 in RPCs; (ii) *Crb1Crb2* cKO with CRB1 and CRB2 ablated in RPCs; (iii) *Crb1*^*KO*^*Crb2*^*ΔimmPRC*^ mice with CRB1 and CRB2 ablated in immature PRCs but remaining levels of CRB2 in MCs and RPCs; and (iv) *Crb1*^*KO*^*Crb2*^*ΔMG*^ mice with CRB1 and CRB2 ablated in MCs but remaining levels of CRB2 in PRCs and RPCs. All four LCA-like models show two consistent characteristics: (i) the integrity of the OLM and retinal lamination is disrupted by ectopic localization of PRCs, and (ii) retinal thickness in the early stage is not significantly reduced or even transiently thickened due to the intermingling of nuclei in ONL and INL, and the ectopic retinal cells in ganglion cell layer (GCL). But as the increased number of apoptotic cells, retina exhibits progressive thinning over time. Retinal dystrophy caused by a single *CRB1* mutation is often limited to one quadrant of the retina. In contrast, additional deletion of CRB2 in the *CRB1* knockout retina exacerbates the retinal phenotype, suggesting that CRB2 putatively acts as a modifying factor of human CRB-associated retinopathies. However, there are four mouse lines with concomitant CRB2 loss in the homozygous *Crb1*^*−/−*^ background considered to be RP models: *Crb1Crb2*^*F/+*^ cKO, *Crb1*^*KO*^*Crb2*^*Low − immPRC*^, *Crb1*^*KO*^*Crb2*^*ΔRods*^, and *Crb1*^*KO*^*Crb2*^*LowMGC*^. The moderate phenotypes of these models seam to contradict conventional cognition. Firstly, the onset of CRE expression after retinogenesis should be a considerable factor. Then, we hypothesize that *CRB2* gene might be a crucial candidate modifier for the severity of CRB1-associated retinopathies, and moderate levels of CRB2 could partially protect against the degeneration caused by CRB1 deficiency. This potential regulatory does not appear to follow a simple linear relationship. CRB-associated dystrophies exist complex genotype-phenotype correlation (Table 1).

**Table 1:** Comparison of CRB–associated mouse models

Mouse line	Expression of CRB1	Expression of CRB2	Mimicking human CRB–associated retinopathy	Predominantly involved quadrants	Retinal phenotype onset	Severity
**Mouse retina lacking CRB1**						
*Crb1*^*rd8*^	Truncated (Truncating the transmembrane and cytoplasmic domains)	Normal	RP	Inferior nasal	P14	++++
*Crb1*^*C249W*^	Truncated (Truncating the extracellular domain)	Normal	RP	NA	8 M	+
*Crb1*^*−/−*^	Ablated	Normal	RP	Inferior temporal	P14	+++++
**Mouse retina lacking CRB2**						
*Crb2*Chx10 cKO	Normal	Ablated in RPCs	RP	Throughout	E18.5	+++++ +
*Crb2*^*ΔimPRC*^	Normal	Ablated in immature PRCs (Remaining CRB2 levels in MCs and RPCs)	RP	Throughout	E15.5	+++++ +
*Crb2*^*ΔRods*^	Normal	Ablated in rods (Remaining CRB2 levels in cones, MCs and RPCs)	RP	Superior	6 M	+
*Crb2*Pdgfrα cKO (*Crb2*^*ΔMG*^)	Normal	Ablated in MCs (Remaining CRB2 levels in PRCs and RPCs)	RP	Periphery	1 M	++
**Mouse retina lacking CRB1 and CRB2**						
*Crb1*^*+/−*^*Crb2* cKO	Reduced	Ablated in RPCs	LCA	Throughout	E15.5	+++++ +++
*Crb1Crb2* cKO	Ablated	Ablated in RPCs	LCA	Throughout	E13	+++++ +++++
*Crb1*^*+/−*^*Crb2*^*F/+*^ cKO	Reduced	Reduced in RPCs	RP	Throughout	3 M	+
*Crb1Crb2*^*F/+*^ cKO	Ablated	Reduced in RPCs	RP	Inferior	P10	+++++
*Crb1*^*KO*^*Crb2*^*Low − immPRC*^	Ablated	Reduced in immature PRCs (And remaining CRB2 levels in MCs and RPCs)	RP	Inferior	1 M	+++
*Crb1*^*KO*^*Crb2*^*ΔimmPRC*^	Ablated	Ablated in immature PRCs (Remaining CRB2 levels in MCs and RPCs)	LCA	Throughout	E15.5	+++++ ++++
*Crb1*^*KO*^*Crb2*^*ΔRods*^	Ablated	Ablated in rods (Remaining CRB2 levels in cones, MCs and RPCs)	RP	Throughout	3 M	+
*Crb1*^*KO*^*Crb2*^*ΔMG*^	Ablated	Ablated in MCs (Remaining CRB2 levels in PRCs and RPCs)	LCA	Periphery	E17	+++++ +++
*Crb1*^*KO*^*Crb2*^*LowMGC*^	Ablated	Reduced in MCs (And remaining CRB2 levels in PRCs and RPCs)	RP	Inferior	3 M	+++++ ++

+ - +++++ +++++: severity ranges from mild to severe

### Rat models mimicking CRB-associated retinopathies

A Brown Norway from Janvier rat strain (BN-J) was reported with a spontaneous in frame insertion-deletion (indel) mutation in exon 6 of *CRB1* gene, which expressing an alternative CRB1^INDEL^ protein [74]. The CRB1^INDEL^ protein was below the detection level in BN-J, which accompanied by a more dispersed distribution of CRB2 [35]. The retinal degeneration was observed from P10 onwards in BN-J retinas, including protrusion of PRCs nuclei into the PRCs segment layers, mislocalization of MCs, and disruption of OLM. Then, with the passing of time, the BN-J retinas developed a progressive lack of retinal lamination with intermingling of nuclei between ONL and INL, retinal cyst and telangiectasia, intraretinal neovascularization, as well as significantly reduced retinal function. This phenotype bears marked resemblance to human macular telangiectasia type 2 (MacTel 2) [74, 75]. Interestingly, the retinal phenotype in BN-J is comparable to that in the mice lacking CRB1 (*Crb1*^*rd8*^, *Crb1*^*C249W*^, and *Crb1*^*−/−*^ mice). However, in mouse models lacking CRB1, the retina degeneration tends to be confined to the inferior quadrant, whereas in the BN-J retinas, it is presented throughout the entire retina and ultimately leads to much more severe retinal dysfunction and vision impairment [27–29, 35].

### Zebrafish models mimicking CRB-associated retinopathies

#### Zebrafish retina lacking Crb1

To analyze the function of Crb1 in zebrafish, Guo et al. [40]. examined the phenotype of the *crb1*^*sa12558*^ nonsense mutant, which carries a C1525T nucleotide exchange in the sixth exon, resulting in a truncated extracellular domain in the middle. Although the loss of Crb1 in zebrafish did not affect the general retinal patterning or overall cone morphology, it weakened the responsiveness of the cones to light. In addition, it resulted in a more significant reduction in the number of PRCs under damaging light irradiation [40]. Furthermore, phylogenetic analysis of vertebrate Crb proteins showed that Crb1 in zebrafish was more closely related to Crb1 in other vertebrate species. In contrast, its specific expression pattern, subcellular localization, and functions were distinct from those of other vertebrate Crb protein homologs [40]. These differences indicate that zebrafish Crb1 may represent neofunctionalization during evolution and remains to be further explored.

#### Zebrafish retina lacking Crb2a

Four mutations affecting the *Crb2a* locus were identified during a large-scale chemical mutagenesis screening of zebrafish embryogenesis: *crb2a*^*m98*^, *crb2a*^*m289*^, *crb2a*^*m298*^, and *crb2a*^*m320*^ [76]. All four mutants produce recessive, fully penetrant, and indistinguishable retinal phenotypes [76].

The *crb2a*^*m98*^ and *crb2a*^*m289*^ carry single base-pair substitutions and introduce stop codons at amino acid positions 493 and 764, respectively, resulting in the loss of transmembrane and cytoplasmic regions and complete loss of function [39].

Earlier studies of *crb2a*^*m98*^ zebrafish have shown that most retinal neurons appear well-differentiated but cannot localize to their normal positions [76, 77].

Most subsequent studies were conducted on *crb2a*^*m289*^ zebrafish, in which PRCs were dispersed all over the retina [36, 77]. Transferring the full-length wild-type *Crb2a* gene into mutant embryos resulted in the formation of PRC rosettes, which could not be adequately replaced by transferring the *Crb2a* gene without the extracellular region (*crb2a*^*ΔEX*^), suggesting that Crb2a endows the PRCs with adhesion properties through its extracellular domain [36, 78].

In Crb2a^ΔEX^; *crb2a*^*m289*^ zebrafish (*crb2a*^*m289*^ mutant embryos expressing the transgenic Crb2a^ΔEX^), the expression of the *Crb2a* cytoplasmic region partially rescued the retinal morphogenesis defects, partially restored the accumulation of adhesion molecules, substantially stabilized its cytoplasmic partner Nagie oko (Nok, MPP5a in zebrafish), and sufficiently inhibited uncontrolled proliferation [78].

In contrast, overexpression of Crb2a^ΔEX^ in wild-type embryos (ZJUOE120; WT zebrafish) competitively impeded the interaction of endogenous Crb2a and Crb2b with Nok, effectively disrupting the integrity of OLM and zonula adherens (ZAs) and the correct localization of endogenous polarity proteins in PRCs [78].

In summary, both the intracellular and extracellular regions of Crb2a are required, but they play distinct roles in guiding the assembly of ZAs. The intracellular region first promotes the apical accumulation of AJs. The extracellular domain then guides the AJ molecules to transform into stable ZAs, thereby guiding retinal morphogenesis [78].

#### Zebrafish retina lacking Crb2b

Knocking down the function of *Crb2b* with morpholino oligonucleotides in embryos resulted in the complete or near-complete loss of its transcript [39]. In addition, although retinal lamination was fully developed in *crb2b* morphants, the IS of PRCs was significantly shorter, indicating that the primary function of Crb2b was to determine the apical surface area of PRCs [39].

The second transcriptional start site in the *Crb2b* gene yields a shorter transcript, *Crb2b-sf*, as opposed to the previous conventional longer form (*Crb2b-lf*) [36]. Crb2b-sf does not contain the first 11 EGF-like repeats; therefore, its extracellular domain is shorter than that of Crb2b-lf [36]. To analyze the function of the extracellular region of Crb2b, Zou et al. [36]. generated a transgenic zebrafish line (abbreviated as *pt108b*) in which the secreted form of the Crb2b-sf extracellular region (Crb2b-sf^− EX^) was primarily produced by blue cones and less frequently by green cones and rods. The enriched Crb2b-sf^− EX^ molecules would compromise the adhesion functions of endogenous Crb2a/Crb2b by competitively binding to their extracellular domains [36]. Additionally, we previously demonstrated that the secreted form of Crb2b-sf^− EX^ in aged *pt108b* retinas increased the density of rods and selectively decreased the density of RGB cones but had no effect on UV cones [79]. In addition, the planar mosaic of surviving cones is strongly disrupted [36, 79]. However, constitutive expression of the Crb2b extracellular region causes RP-like degeneration, but the integrity of the OLM is largely intact without PRCs mislocated into the GCL or INL [36]. These ectopic variations were not observed in young adult *pt108b* retinas [79]. Together, the expression of Crb2b-sf^− EX^ selectively interferes with PRC maintenance but does not affect their development. Considering that this type of selective RGB cone death has not been observed in CRB-associated mouse models, our previous study’s data suggest that the *pt108b* zebrafish line can provide a valuable new tool for gaining insights into the mechanism by which Crb proteins maintain RGB cones, UV cones, and rods. This contributes to the further investigation of CRB-associated retinopathies that target specific cone subtypes [79].

The *Crb2b* knockout zebrafish line, *crb2b*^*ZJUKO201*^, shows well-developed retinal lamination with proper localization of retinal cells and intact OLM [80]. The loss of Crb2b resulted in the ectopic expansion of Crb2a in the PRC IS in the planar plane and the gradual disruption of the cone mosaic accompanied by the elongated eye axis [80]. Crb proteins mediate cell–cell adhesion through their extracellular domains [36]. In wild-type retinas, Crb2b regulates the planar distribution of Crb2a and stabilizes the cone mosaic through heterophilic interactions (Crb2a–Crb2b). In contrast, either Crb2a or Crb2b can regulate homophilic interactions (Crb2a–Crb2a or Crb2b–Crb2b) when there is only one homolog [80].

Altogether, all CRB-associated zebrafish models to date have shown mild RP-like phenotypes: *crb1*^*sa12558*^, *crb2a*^*m98*^, *crb2a*^*m289*^, *crb2a*^*m298*^, *crb2a*^*m320*^, Crb2a^ΔEX^; *crb2a*^*m289*^, ZJUOE120; WT, *crb2b* morphants, *pt108b*, and *crb2b*^*ZJUKO201*^. Among them, the models lacking Crb2a showed relatively more severe morphogenesis defects, including disruption of OLM. Other models exhibited two consistent characteristics: (i) the retinal lamination was developed with largely intact integrity of the OLM, and (ii) the PRCs showed mildly abnormal morphology without ectopic localization. In addition, *crb1*^*sa12558*^ and *pt108b* mutants showed selective death of cone subtypes (B and RGB cones, respectively) (Table 2). The various fates of PRC subtypes indicate that their maintenance and survival mechanisms may be different and not interdependent in zebrafish.

**Table 2:** Comparison of CRB–associated zebrafish models

Zebrafish line	Expression of Crb	OLM disruption	Abnormal retinal lamination	Rosettes (Aggregation of PRCs)	Severity
**Zebrafish retina lacking Crb1**					
*crb1*^*sa12558*^	Truncated Crb1 (Truncating the extracelullar domain)	No	No	No	I
**Zebrafish retina lacking Crb2a**					
*crb2a*^*m98*^, *crb2a*^*m289*^, *crb2a*^*m298*^, *crb2a*^*m320*^	Truncated Crb2a (Truncating the transmembrane and cytoplasmic domains)	Yes	Yes	No	V
Crb2a^ΔEX^; *crb2a*^*m289*^	Transferred *crb2a*^*ΔEX*^ into *crb2a*^*m289*^ embryos	Yes (Partially rescued AJs assembly)	Yes (Partially rescued retinal lamination defects)	Yes	IV
ZJUOE120; WT	Transferred *crb2a*^*ΔEX*^ into wild type embryos	Yes	Yes	Yes	III
**Zebrafish retina lacking Crb2b**					
*crb2b* morphant	Knocked down the function of Crb2b	No	No	No	II
*pt108b*	Transferred a secreted form of Crb2b–sf extracellular domain	No	No	No	II
*crb2b*^*ZJUKO201*^	Ablated Crb2b	No	No	No	II

I - V: severity ranges from mild to severe

Cone mosaics of various vertebrates are assembled in distinct patterns [81], whereas cones in adult zebrafish are assembled into an ordered rectangular lattice mosaic [36, 82]. Considering the responsibilities of cones in perceiving color and daylight vision, it is speculated that planar cone mosaics may be related to color perception and visual acuity [83]. This significantly disrupted mosaic indicates that Crb proteins play vital roles in establishing planar cell polarity. Furthermore, the evaluation of visual function should include an examination of color perception and not be limited to ERG.

Zebrafish is a popular model organism for investigating the functions of vertebrate genes and displays significant physiological homology to humans (up to 87%) [84]. The morphological development of the zebrafish retina is highly conserved, consisting of 10 layers and sharing the same neuron composition as the human retina [85–87]. The retina of zebrafish is dominated by cones (60% cones and 40% rods), as is the central retina of humans [75, 88]. In contrast, the retina of nocturnal rodents (such as rats and mice) is dominated by rods (97% rods and 3% cones), which is similar to the peripheral retina of humans [88, 89]. Therefore, the zebrafish retina has unique and excellent advantages in exploring the pathogenesis of human retinal degeneration diseases.

### Human models mimicking CRB-associated retinopathies

The iPSC-derived human retinal organoids recapitulate the intricate process of retinogenesis by forming well-defined retinal laminations with characteristic markers. These humanized retinal models provide the means for generating retinal cells from patients with inherited retinal dystrophies (IRDs) carrying disease-causing mutations [90], and are expected to provide a promising alternative or additional/pre-screening tool to current animal models for evaluating transgene expression and biological activity [91].

So far, multiple CRB1 patient-derived iPSC lines were generated and differentiated into CRB1 patient-derived retinal organoids, showing different variants of CRB1 protein [42, 92–95]. LEIi006-A carries c.1892 A > G p.(Tyr631Cys) and c.2548 G > A p.(Gly850Ser) heterozygous mutations [95, 96]; LEIi016-A and LEIi016-B carry c.2555 T > C p.(Ile852Thr) and c.3014 A > T p.(Asp1005Val) heterozygous mutations [94, 97, 98]; LUMC0116iCRB carries c.3122 T > C p.(Met1041Thr) homozygous mutations; LUMC0117iCRB carries c.1892 A > G p.(Tyr631Cys) and c.2983 G > T p.(Glu995*) heterozygous mutations; LUMC0128iCRB carries c.2843 G > A p.(Cys948Tyr) and c.3122 T > C p.(Met1041Thr) heterozygous mutations [42] (Table 3). All of these CRB1 iPSC lines are able to differentiate into retinal organoids with typical three retinal layers, which are comparable to healthy retinal organoids: a ganglion cell layer, a neurobasal layer (NBL), and an ONL [42, 92–95].

**Table 3:** Comparison of CRB–associated iPSC lines

iPSC line	Description	Homozygous or heterozygous		References
**Human retinal organoid lacking CRB1**				
LEIi006-A	c.1892 A > G p.(Tyr631Cys); c.2548 G > A p.(Gly850Ser)	Heterozygous		[95, 96]
LEIi016-A, LEIi016-B	c.2555 T > C p.(Ile852Thr); c.3014 A > T p.(Asp1005Val)	Heterozygous		[94, 97, 98]
LUMC0116iCRB	c.3122 T > C p.(Met1041Thr)	Homozygous		[42]
LUMC0117iCRB	c.1892 A > G p.(Tyr631Cys); c.2983 G > T p.(Glu995*)	Heterozygous		[42]
LUMC0128iCRB	c.2843 G > A p.(Cys948Tyr); c.3122 T > C p.(Met1041Thr)	Heterozygous		[42]
*CRB1*^KO^ lines (CL19, CL26, CL72)	c.500del p.(Ser44Serfs*)	Homozygous		[99]
**Human retinal organoid lacking CRB1 and CRB2**				
*CRB1*^KO^*CRB2*^+/−^ lines (CL4, CL9)	*CRB1*: c.500del p.(Ser44Serfs*)	Homozygous		[99]
	*CRB2*: c.576_598del p.(Cys193Argfs*)	Heterozygous		
*CRB1*^KO^*CRB2*^+/−^ lines (CL17)	*CRB1*: c.498_507delinsTGCC p.(Ser44Lysfs*)	Homozygous		[99]
	*CRB2*: c.583_584del p.(His195Trpfs*)	Heterozygous		

In LUMC0116iCRB, LUMC0117iCRB, and LUMC0128iCRB, the CRB1 variant proteins (CRB1^Met1041Thr/Met1041Thr^; CRB1^Tyr631Cys/Glu995*^; CRB1^Cys948Tyr/Met1041Thr^) localized at the SARs above AJs, similar to the healthy control lines, but showed diminished levels with a more curved and broadened distribution pattern. In addition, there were also a small number of CRB1 variant proteins mislocalized in the apical area of the NBL and in the ONL [42, 93]. These three retinal organoids developed small but frequent disruptions of CRB complex at the OLM on differentiation day (DD) 180, permitting disruptions at OLM with local displacement of PRCs columns [42]. Then, a moderate decrease in the number of PRCs and ONL thickness was observed at a later time (DD210) [93].

Moreover, Boon et al. [99]. generated three *CRB1*^*KO*^ (CL19, CL26, CL72) and three *CRB1*^*KO*^*CRB2*^*+/−*^ (CL4, CL9, CL17) iPSC lines using CRISPR-Cas9 technology: CL19, CL26, and CL72 carry c.500del p.(Ser44Serfs*) homozygous mutations for *CRB1*; CL4 and CL9 carry c.500del p.(Ser44Serfs*) homozygous mutations for *CRB1*, and c.576_598del p.(Cys193Argfs*) heterozygous mutations for *CRB2*; CL17 carries c.498_507delinsTGCC p.(Ser44Lysfs*) homozygous mutations for *CRB1*, and c.583_584del p.(His195Trpfs*) heterozygous mutations for *CRB2* (Table 3). The *CRB1*^*KO*^ and *CRB1*^*KO*^*CRB2*^*+/−*^ retinal organoids showed a similar outer retinal phenotype as the *CRB1* patient-derived retinal organoids: a significant increase in the number of PRC nuclei above OLM, a significant decrease in the number of PRC nuclei in a row and ONL thickness [42, 93, 99]. In addition, a less defined alignment of inner retina was observed in the *CRB1*^*KO*^ and *CRB1*^*KO*^*CRB2*^*+/−*^ retinal organoids [99]. This inner retinal phenotype was previously not observed in the *CRB1* patient-derived retinal organoids [42, 93]. In summary, the complete loss of CRB1 in *CRB1*^*KO*^ and *CRB1*^*KO*^*CRB2*^*+/−*^ retinal organoids leads to more extensive degeneration of both inner and outer retina, mimicking a mild form of LCA, whereas the CRB1 patients-derived retinal organoids express reduced level of CRB1 variants and show only degeneration of outer retina.

The numerous human-derived CRB1 variants and artificially designed CRB clones have expanded our understanding of the pathogenesis and provided direct guidance for future gene therapies.

### Prospective therapeutic options for CRB-associated retinopathies

Up to now, there is no available treatment for CRB-associated retinopathies. The Food and Drug Administration (FDA) approved an adeno-associated viral (AAV) mediated gene therapy (Luxturna) for IRDs with *RPE65* mutations, which has attracted emerging interests of gene augmentation therapies using AAV vectors [100]. Today, AAVs are the leading platform for in vivo delivery of gene therapies because of their low immunogenicity and cytotoxicity, and the potential to target specific tissues following systemic administration [101]. In addition, different AAV capsids display distinct cell tropisms, making it enable to target specific retinal subpopulations [101]. However, developing an AAV mediated gene therapy for CRB-associated retinopathies is more challenging due to the large size of CRB1 and CRB2 cDNA (4.4 kb and 3.9 kb, respectively) which approach the AAV packaging limit (~ 5.0 kb, including inverted terminal repeats), a disorder affecting three different cell types (cones, rods, and MCs), and the complex functions of CRB as structural and signaling transmembrane proteins [101–103].

Previous mouse gene augmentation studies using AAV expressing human CRB2 have shown both morphological and functional rescue in CRB-associated RP mouse models (*Crb1Crb2*^*F/+*^ cKO, *Crb2*Chx10 cKO, and *Crb1*^*KO*^*Crb2*^*LowMGC*^), suggesting the great potential of AAV mediated gene augmentation therapies for CRB patients [71, 104]. However, cross-species differences in AAV tropism have been previously highlighted, indicating the importance of using human-derived models for gene augmentation therapies [105, 106]. Transduction studies showed that AAV5 is more potent than AAV9 for infecting PRCs in cadaveric human retinal explants, AAV5 and ShH10Y445F are more efficient than AAV9 for infecting retinal organoids, and human PRCs are efficiently transduced only in the presence of PRC segments [42]. Then, Boon et al. [93]. demonstrated a partially restored phenotype after AAV mediated human *CRB1* or *CRB2* augmentation therapies in CRB1 patient-derived retinal organoids. The ONL thickness and the number of PRC nuclei in a row were significantly improved after treatment when analyzed at DD120, suggesting the long-term effects of the gene augmentation therapy. Similarly, AAV mediated human *CRB2* also improved the outer retinal phenotype in *CRB1*^*KO*^ retinal organoids [99]. Altogether, these data provide essential information for future gene augmentation therapies for CRB-associated retinopathies.

Gene editing may be another viable strategy for the amelioration of CRB-associated retinopathies [107, 108]. Due to the various CRB1 isoforms and their cell-type-specific localizations in vertebrate species, a therapeutic approach such as AAV mediated gene augmentation is complicated and unclear [25]. Motta et al. [109]. found a direct relation between mutation effect and phenotype severity in CRB1 patients despite the deficiency of clear genotype-phenotype correlation. These data highlight the need for developing a CRB1 isoform independent treatment modality, such as precise gene editing strategy applying clustered regularly interspaced short palindromic repeats (CRISPR). Base editing can modify single-strand DNA through specific and single nucleotide alterations, whereas prime editing can directly re-write new genetic information into a specified DNA site [110–112]. The Leiden Open Variation Database (LOVD) was searched for previously reported *CRB1* variants (https://databases.lovd.nl/shared/variants/CRB1/unique; accessed on May 14, 2024). The top 10 most common variants accounts for 32.96% of all pathogenic alleles, while the most prevalent *CRB1* mutation is c.2843G > A p.(Cys948Tyr), accounting for 12.11% of all pathogenic alleles (Table 4). Costa et al. [107]. evaluated base and prime editing strategies for the 10 most prevalent mutations (including variants from #1 to #10.1). Briefly, base editing is suitable for 6 of the 10 variants, whereas prime editing is feasible for all 10 variants. Developing a proper gene editing approach for c.2843G > A p.(Cys948Tyr) would be attractive owing to its prevalence among patients. This proof-of-concept evaluation shows the potential promise for the clinical application of *CRB1* gene editing, particularly to circumvent the complexity of CRB1 isoforms.

**Table 4:** The 10 most frequent *CRB1* variants in the LOVD

Variant #	cDNA change	Protein change	Exon	Reported alleles (number)	Proportion of alleles (%)
1	c.2843G > A	p.(Cys948Tyr)	9	262	12.11
2	c.2234 C > T	p.(Thr745Met)	7	74	3.42
3	c.2401 A > T	p.(Lys801*)	7	73	3.37
4	c.2290 C > T	p.(Arg764Cys)	7	67	3.10
5	c.498_506del	p.(Ile167_Gly169del)	2	47	2.17
6	c.2688T > A	p.(Cys896*)	8	45	2.08
7	c.613_619del	p.(Ile205Aspfs*13)	2	34	1.57
8	c.1576 C > T	p.(Arg526*)	6	30	1.39
9	c.3307G > A	p.(Gly1103Arg)	9	21	0.97
10.1	c.614T > C	p.(Ile205Thr)	2	20	0.92
10.2	c.3299T > C	p.(Ile1100Thr)	9	20	0.92
10.3	c.3676G > T	p.(Gly1226*)	9	20	0.92
**Total**				**713**	**32.96**

More recently, Peng et al. [113]. innovatively revealed a new theory that *CRB1* mutations in *Crb1*^*rd8*^ mice lead to failure of both colonic and retinal epithelial barrier integrity, permitting bacterial translocation from the leaky gut to the outer retina where they cause secondary retinal degeneration. Either systemic broad-spectrum antibiotic therapy or restoration of intestinal *CRB1* expression could mitigate this retinal damage. These results break the traditional viewpoint that CRB-associated retinopathies are local ocular pathologies, and provide evidence that may be a kind of multiorgan disorder. Antimicrobial agents surprisingly have the potential to treat this devastating blinding disease.

## Conclusions

In the past two decades, many studies have demonstrated the pathogenesis of CRB-associated retinopathies. Most of the knowledge acquired resulted from the analysis of multiple animal models. Several rodent models lacking one or both *CRB1*/*CRB2* homologs present retinal dystrophy of different severities, indicating the functional redundancy and complex gene modifying system among CRB proteins. Zebrafish models lacking *Crb* homologs generally show milder phenotypes, which may be related to the potentially more significant functional overlap of more subtypes of Crb. Therefore, it is necessary to cross different zebrafish lines to further investigate whether the loss of more homologs aggravates the phenotype. Human-derived retinal models can avoid differences in gene expression and retinal development patterns between clinical patients and animal models, which are of great interest to understand the complete pathogenesis of CRB-associated retinopathies. Nevertheless, mutations in *CRB* lead to diverse severities of retinal degenerations ranging from the early-onset RP to the more severe LCA. The deficiency of clear genotype-phenotype correlation is very likely due to complex genetic regulatory system. Further genetic investigations on CRB-associated patients may be helpful to provide more in-depth understanding.

The recent clinical advances in retinal gene augmentation therapies show that AAV can safely and precisely deliver genes to retina. The promising initial results from mouse models and human-derived models using AAV mediated *CRB* therapy suggest that CRB-associated RP may potentially be treatable. However, retinas from LCA patients may have already irreversible impairment at birth, so it is more challenging to ameliorate this kind of dystrophies. In addition, how to overcome the limited packaging capacity while retaining the efficient targetability remains a tough question worth further exploration. In short, translating retinal gene augmentation therapy from pre-clinical research into clinical application is still a lengthy process.

The proposal of “translocated-bacteria-dependent retinal degeneration” provides a new perspective for pathogenesis and treatment of CRB-associated retinopathies. This proof of principle suggests that retinal degeneration is not local ocular pathologies, as has long been considered, but a multiorgan disorder that may be mitigated by antimicrobial treatment.

Although gene augmentation therapies and antibiotic treatments for CRB-associated retinopathies are still in the early stages, these recent steps have made significant contributions to rescuing this devastating blinding disease.

## Data Availability

No datasets were generated or analysed during the current study.

## Abbreviations

CRB: Crumbs protein
RP12: Retinitis pigmentosa type 12
LCA8: Leber congenital amaurosis type 8
PALS1: Protein Associated with Lin Seven 1
PATJ: PALS1-associated tight junction protein
MUPP1: Multi-PDZ domain Protein 1
EGF: Epidermal growth factor
EPB4.1L5: Erythrocyte Protein Band 4.1-like 5
lrCaptureSeq: Long-read capture sequencing
SARs: Subapical regions
AJs: Adherens junctions
MCs: Müller glial cells
PRCs: Photoreceptor cells
OLM: Outer limiting membrane
IS: Inner segments
OS: Outer segments
RPE: Retinal pigment epithelium
RPCs: Retinal progenitor cells
iPSC: Induced pluripotent stem cell
NRF2: Nuclear factor E2-related factor 2
AMD: Age-related macular degeneration
CNV: Choroidal neovascularization
MTHFR: Methylenetetrahydrofolate reductase
Cygb: Cytoglobin
M: Months
Pttg1: Pituitary tumor transforming gene 1
INL: Inner nuclear layer
ONL: Outer nuclear layer
cKO: Conditional knockout
E: Embryonic day
P: Postnatal day
Pdgfrα: Platelet-derived growth factor receptor, alpha polypeptide
ERG: Electroretinography
DL-AAA: DL-α-aminoadipate acid
GCL: Ganglion cell layer
BN-J: Brown Norway from Janvier
Indel: Insertion-deletion
MacTel 2: Macular telangiectasia type 2
Nok: Nok
ZAs: Zonula adherens
IRDs: Inherited retinal dystrophies
NBL: Neurobasal layer
DD: Differentiation day
FDA: Food and Drug Administration
AAV: Adeno-associated viral
CRISPR: Clustered regularly interspaced short palindromic repeats
LOVD: Leiden Open Variation Database
NA: Not available
